# Integrated Clinical and Molecular Profiling of Fetal Growth Disorders in the First Trimester

**DOI:** 10.3390/ijms27104192

**Published:** 2026-05-08

**Authors:** Natalia Starodubtseva, Alisa Tokareva, Natalia Frankevich, Alexey Kononikhin, Anna Bugrova, Maria Indeykina, Evgenii Kukaev, Anna Derenko, Vladimir Frankevich, Evgeny Nikolaev, Gennady Sukhikh

**Affiliations:** 1 V.I. Kulakov National Medical Research Center for Obstetrics Gynecology and Perinatology, Ministry of Healthcare of Russian Federation, Oparina Str. 4, 117997 Moscow, Russia; a_tokareva@oparina4.ru (A.T.); n_frankevich@oparina4.ru (N.F.); as.kononikhin@gmail.com (A.K.); anna.bugrova@gmail.com (A.B.); e_kukaev@oparina4.ru (E.K.); derenkoaa@medudp.ru (A.D.); g_sukhikh@oparina4.ru (G.S.); 2 Moscow Center for Advanced Studies, 123592 Moscow, Russia; 3 The Center for Bio- and Medical Technologies, 121205 Moscow, Russia; mariind@yandex.ru (M.I.); ennikolaev@gmail.com (E.N.); 4 Emanuel Institute of Biochemical Physics, Russian Academy of Sciences, Kosygina Str. 4, 119334 Moscow, Russia; 5 V.L. Talrose Institute for Energy Problems of Chemical Physics, N.N. Semenov Federal Research Center for Chemical Physics, Russian Academy of Sciences, Leninsky Av.38/2, 119334 Moscow, Russia; 6 Department of Obstetrics, Gynecology, Perinatology and Reproductology, Institute of Professional Education, Federal State Autonomous Educational Institution of Higher Education I.M. Sechenov First Moscow State Medical University of the Ministry of Health of the Russian Federation, Trubetskaya Str. 8/2, 119991 Moscow, Russia

**Keywords:** gestational diabetes mellitus, fetal macrosomia, intrauterine growth restriction, proteomics, pregnancy, biomarkers, placental dysfunction, complement system

## Abstract

This prospective study evaluated first-trimester markers in pregnancies with isolated and combined forms of fetal growth disorders and gestational diabetes mellitus (GDM). Among 1869 screened women, the analysis included 83 controls, 55 GDM, 22 isolated intrauterine growth restriction (iIUGR), and 33 isolated large-for-gestational-age (iLGA) cases, with GDM subgroups stratified by fetal growth (GDM with normal fetal weight, GDM + IUGR, and GDM + LGA). First-trimester clinical and routine biochemical parameters were recorded, and serum concentrations of 80 proteins were measured using targeted LC-MRM-MS proteomics. Different trajectories emerged: IUGR phenotypes showed low PAPP-A/PlGF and high TSH (*p* < 0.01), indicating early placental insufficiency, while macrosomia showed opposite trends. GDM + IUGR represented the most severe “double hit” phenotype (lowest PlGF, earliest delivery), whereas GDM + LGA showed increased umbilical artery resistance despite excessive growth, suggesting endothelial dysfunction. Targeted proteomics revealed characteristic signatures: iIUGR featured low complement (*C4A|C4B*) and IGF proteins (*IGFALS*, *IGFBP3*) versus GDM and iLGA (*p* < 0.001); GDM + IUGR showed elevated *PZP* and *CD5L* versus iIUGR (*p* < 0.05); GDM + LGA was marked by high *C4BPA* and low *RBP4*, *SERPINA7* versus iLGA (*p* < 0.05). Complement and IGF pathways were consistently implicated. Machine learning achieved 77% sensitivity for IUGR prediction using clinical parameters and 88% sensitivity for LGA prediction using proteomic data. These findings demonstrate that fetal growth disorders represent pathophysiologically unique entities detectable in the first trimester, enabling early risk stratification and personalized management.

## 1. Introduction

Gestational diabetes mellitus (GDM) and fetal growth disorders, including intrauterine growth restriction (IUGR) and fetal macrosomia (LGA), represent some of the most common pregnancy complications, associated with significant risks of adverse perinatal outcomes and long-term metabolic disturbances in offspring [1,2]. Traditionally, GDM, IUGR, and LGA have been viewed as distinct clinical entities with different etiologies: GDM is linked to maternal metabolic disturbances, IUGR to placental insufficiency, and LGA to excessive fetal nutrition and constitutional factors [3]. It is important to distinguish between the terms fetal macrosomia and large for gestational age (LGA). Although these concepts are often used interchangeably, they are not identical: fetal macrosomia is typically defined as an absolute birth weight exceeding 4000 g or 4500 g, regardless of gestational age, whereas LGA is defined as a birth weight above the 90th percentile for a given gestational age and sex. Both conditions indicate excessive fetal size, but their etiology, risk profiles, and management strategies may differ.

In clinical practice, combined forms of these pathologies—GDM with IUGR or LGA—are encountered relatively infrequently. However, when they do occur, these combined conditions are associated with markedly worse pregnancy outcomes and present considerable diagnostic and therapeutic challenges [4]. The two main phenotypes of GDM associated with fetal macrosomia (GDM + FM) and with fetal growth restriction (GDM + FGR) represent distinct “metabolic poles” of the same pathology, shaping fundamentally different trajectories of antenatal development. Whereas in GDM + FM maternal hyperglycemia results in excessive nutrient supply and fetal hyperinsulinemia, in GDM + FGR the same metabolic conditions paradoxically coincide with placental dysfunction that limits nutrient availability. Clinically, this means that behind a single diagnosis of GDM may lie risks of birth trauma and neonatal hypoglycemia, as well as chronic hypoxia syndrome with growth restriction, requiring fundamentally different strategies for monitoring the pregnant woman and the fetus.

The mechanisms driving these rare but severe phenotypes remain elusive, and it is uncertain whether they represent simple comorbidity or distinct pathophysiological entities with unique molecular signatures. Importantly, while first-trimester screening programs incorporating maternal characteristics and biochemical markers (including pregnancy-associated plasma protein A, PAPP-A and placental growth factor, PlGF) have demonstrated utility in predicting preeclampsia and IUGR, their ability to identify isolated and combined fetal growth disorders in pregnancies complicated by GDM remains to be established [5,6].

Recent advances in proteomic technologies provide powerful tools to investigate the complex interactions between maternal metabolism, placental function, and fetal growth. In particular, the identification of specific protein panels in first-trimester maternal blood may both deepen our understanding of pathogenesis and enable early differentiation of these phenotypes for personalized pregnancy management [7,8]. To address this, the present study performed a comprehensive analysis of clinical and obstetric history data, first-trimester routine biochemical markers, and serum proteomic profiles to identify features that distinguish isolated and GDM-combined forms of IUGR and fetal macrosomia.

## 2. Results

### 2.1. Clinical and Biochemical Profiles of the Study Cohorts

A comparative analysis was first conducted across four clinical groups: a control group of women with uncomplicated pregnancies (*n* = 83), a group with GDM (*n* = 55), a group with isolated IUGR (iIUGR, *n* = 22), and a group with isolated LGA (iLGA, *n* = 33). Within the GDM group, three additional subgroups were defined based on fetal growth outcomes: GDM with normal fetal weight (GDM + NW, *n* = 39), GDM with IUGR (GDM + IUGR, *n* = 8), and GDM with macrosomia (GDM + LGA, *n* = 8). The limited size of the GDM + IUGR and GDM + LGA subgroups reflects the relatively low incidence of these combined phenotypes, each accounting for approximately 15% of GDM pregnancies in our cohort.

Analysis of obstetric history revealed a higher prevalence of prior spontaneous miscarriages, recurrent pregnancy loss, and previous IUGR among women with isolated IUGR. The frequency of spontaneous miscarriage in this group was 32%, compared to 5% in the control group (*p* = 0.006) and 5% in the iLGA group (*p* = 0.04). Recurrent pregnancy loss occurred in 14% of the iIUGR group versus 0% in controls (*p* = 0.04), and a history of IUGR was also noted in 14% of cases, significantly higher than in other groups (*p* < 0.001) (Table S1). These findings suggest the presence of chronic, underlying factors, likely of maternal vascular origin, aligning with the theory that IUGR often stems from long-standing endothelial dysfunction (e.g., thrombophilia, antiphospholipid syndrome, chronic hypertension) that may manifest prior to the current pregnancy [5,9].

A high prevalence of prior macrosomia was observed in both the iLGA group (36% vs. 1% in controls, *p* < 0.001; vs. 0% in iIUGR, *p* = 0.02) and the GDM + LGA subgroup (50% vs. 8% in isolated GDM, *p* = 0.004) (Tables S1 and S2, Figure S1). This underscores the role of constitutional factors (genetically predisposed large fetus) and/or undiagnosed carbohydrate metabolism disorders (prediabetes, obesity) in previous pregnancies, contributing to recurrent macrosomia [10].

Maternal age and metabolic status emerged as significant risk factors. Women with GDM were older (34.7 (31.7; 36.3) years vs. 30.3 (27.4; 32.7) years in controls, *p* < 0.001) and had a higher BMI (23.23 (21.45; 27.27) kg/m^2^ vs. 21.22 (19.18; 23.01) kg/m^2^ in controls, *p* < 0.001) (Figure S1). Obesity was more frequent in the GDM group (18%, *p* = 0.002). Maternal obesity and age-related metabolic changes are well-established risk factors for both GDM and macrosomia [11,12].

Analysis of first-trimester routine serum biomarkers revealed distinct patterns across the study groups. Pregnancies complicated by IUGR—whether isolated IUGR or with concomitant gestational diabetes (GDM + IUGR)—exhibited a characteristic profile: elevated maternal thyroid-stimulating hormone (TSH) and reduced levels of pregnancy-associated plasma protein A (PAPP-A) and placental growth factor (PlGF) (Tables S1 and S2).

Specifically, TSH levels were significantly higher in the iIUGR group (2.16 (1.85;3.2) mIU/L) compared to both controls (1.79 (1.24; 2.24) mIU/L, *p* = 0.04) and the iLGA group (1.15 (0.84; 1.76) mIU/L, *p* < 0.001). Similarly, the GDM + IUGR group showed elevated TSH (2.28 (2.05; 2.860 mIU/L) relative to GDM + NW (1.72 (1.23; 2.14) mIU/L, *p* = 0.03) and GDM + LGA (0.9 (0.72; 1.4) mIU/L, *p* < 0.001).

Conversely, PAPP-A levels were reduced in the iIUGR group (1.92 (0.91; 2.24) mIU/L) when compared to controls (3.09 (2.08; 4.59) mIU/L, *p* < 0.001), the GDM group (2.84 (1.9; 4.26) mIU/L, *p* = 0.01), and the iLGA group (3.1 (2.23; 4.39) mIU/L, *p* = 0.002). A similar pattern was observed for PlGF, which was lower in iIUGR (18.61 (12.13; 22.6) pg/mL) than in controls (25.79 (19.9; 32.46) pg/mL, *p* < 0.001), the GDM group (27.69 (16.56; 35.46) pg/mL, *p* = 0.001), and the iLGA group (23.32 (21.6; 27.67) pg/mL, *p* = 0.03).

The GDM + IUGR subgroup exhibited the most pronounced biomarker alterations and adverse clinical outcomes (Table S2, Figure S1). This group had the lowest PlGF levels (14.49 (13.19;16.99) pg/mL), which were significantly reduced relative to GDM + NW (*p* = 0.004) and comparable to those in the iIUGR group (18.61 (12.13; 22.6) pg/mL, *p* = 0.48) (Tables S2 and S3). Clinically, this subgroup experienced the earliest deliveries (37.2 (35.48; 37.55) weeks, *p* < 0.001), the lowest birth weights (1983 (1828; 2106.75) g, *p* < 0.001), and elevated pre-delivery AST levels (27.5 (23.9; 32.4) U/L, *p* < 0.001) compared to other GDM-associated subgroups (Table S2).

In contrast to the IUGR groups, pregnancies with macrosomia displayed an opposing biomarker trend. TSH levels were significantly lower in the GDM + LGA group (0.9 [0.72–1.4] mIU/L) than in the GDM group with normal fetal weight (1.72 [1.23–2.14] mIU/L, *p* = 0.04) (Table S2).

Doppler ultrasound prior to delivery revealed distinct hemodynamic profiles across the study groups (Tables S1, S3 and S4). In iIUGR, the highest umbilical artery pulsatility index (PI, 0.92 (0.82; 1.08), *p* < 0.001) was observed, along with the lowest cerebroplacental ratio (CPR: 1.6 [1.23–1.8], *p* < 0.001) (Table S1). Compared to iIUGR, the GDM + IUGR group showed comparable uterine artery PI (1.92 (1.68;2.09) vs. 1.97 (1.52;2.36), *p* = 0.62), umbilical artery PI (0.82 (0.67; 0.94) vs. 0.92 (0.82; l.08), *p* = 0.21), and CPR (1.37 (1.19; 1.65) vs. 1.6 (1.23; 1.8), *p* = 0.5) (Table S3).

The iLGA group demonstrated the lowest umbilical artery PI (0.69 (0.62; 0.78), *p* < 0.001) and the highest CPR (2.03 (1.82;2.33), *p* < 0.001) relative to the other groups (Table S1). Comparison between iLGA and GDM + LGA revealed significantly higher umbilical artery PI in the GDM + LGA group (0.84 [0.82–0.92] vs. 0.69 [0.62–0.78], *p* = 0.001) (Table S4).

Analysis of delivery and neonatal outcomes revealed the highest rates of preterm birth in the iIUGR (36%, *p* < 0.001) (Table S1) and GDM + IUGR (38%, *p* < 0.001) (Table S2) groups. Cesarean section rates were also elevated in groups with fetal pathology: 59% in iIUGR, 75% in GDM + IUGR, and 45% in isolated GDM, compared to 11% in controls.

### 2.2. Proteome Profiling

To identify early pregnancy protein signatures associated with different pregnancy complications, a multiplex proteomic analysis was performed on first-trimester serum samples obtained from routine prenatal screening. Targeted proteomic analysis quantified 85 proteins that met predefined quality control criteria (R^2^ > 0.99, with precision and accuracy < 20% in at least five of seven standard levels and in more than 66% of quality control (QC) samples; Table S5) and were qualified in more than 50% analytical samples.

Following normalization, five proteins were excluded due to a coefficient of variation (CV) > 20% across QC and calibration standard samples: Hyaluronan-binding protein 2, Ig mu chain C region, Inter-alpha-trypsin inhibitor heavy chain H2, H1, and H4. This resulted in 80 proteins included in the final analysis (Table S6). After normalization to multiples of the median (MoM) and adjustment for clinical parameters, Alpha-2-HS-glycoprotein (*AHSG*) exhibited the lowest level (0.70 (0.44; 0.92) MoM), while complement C4 (*C4A|C4B*) showed the highest level (1.16 (0.84; 1.56) MoM) (Table S7, Figure S2). Regard-ing inter-individual variation, Alpha-2-antiplasmin (*SERPINF2*) was the most stable protein, with a confidence interval width of 0.33 MoM, whereas Pregnancy zone protein (*PZP*) was the least stable, with a confidence interval width of 3.82 MoM (Table S7, Figure S1).

**Figure 1:**
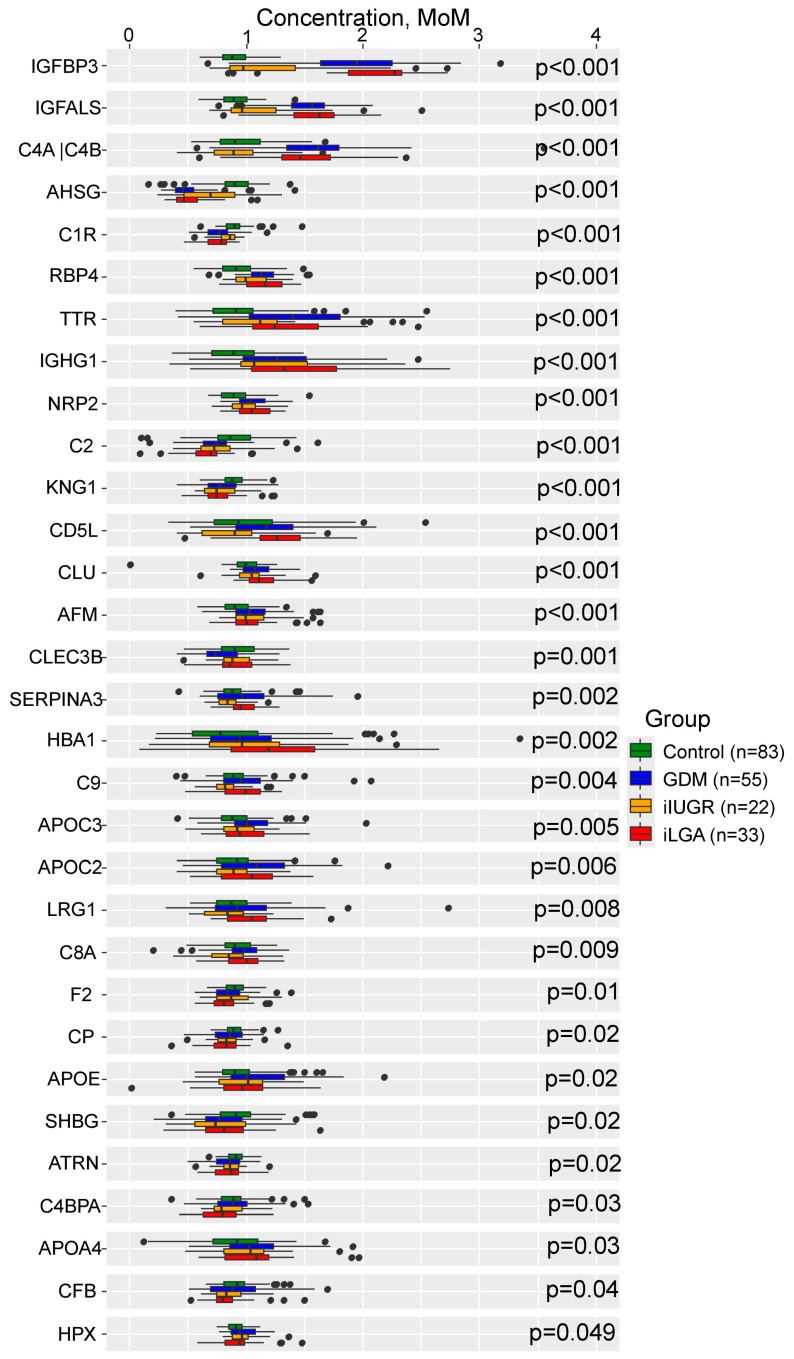
Differential protein expression across clinical groups. Box plots show median levels with interquartile ranges and outliers (dots) for proteins exhibiting statistically significant differences among the four study groups: control (*n* = 83), iIUGR (*n* = 22), GDM (*n* = 55), and iLGA (*n* = 33). Protein levels are expressed as multiples of the median (MoM) after adjustment for clinical parameters. *p*-values were calculated using the Kruskal–Wallis test for overall group comparisons.

Thirty-one proteins (39%) demonstrated statistically significant differences among the four clinical groups (Table S8, Figure 1). Insulin-like growth factor-binding protein 3 (*IGFBP3*), Insulin-like growth factor-binding protein complex acid labile subunit (*IG-FALS*), and *C4A|C4B* were significantly lower in the control and iIUGR groups compared to the GDM and iLGA groups (*p* < 0.001). *AHSG* and complement C1r subcomponent (*C1R*) were significantly higher in the control and iIUGR groups than in the GDM and iLGA groups (*p* < 0.001). Afamin (*AFM*), Ig gamma-1 chain C region (*IGHG1*), and Retinol-binding protein 4 (*RBP4*) were significantly lower in the control group compared to all other groups (*p* < 0.001), while complement C2 (*C2*) and Kininogen-1 (*KNG1*) were significantly higher in the control group than in all other groups (*p* < 0.001).

### 2.3. PLS-DA and OPLS Models for Discrimination of GDM and IUGR Subgroups

To better understand the early serum protein signatures associated with different pregnancy complications, a series of multivariate analyses was performed comparing clinical subgroups. A series of multivariate analyses was then conducted to compare clinical subgroups.

**Figure 2:**
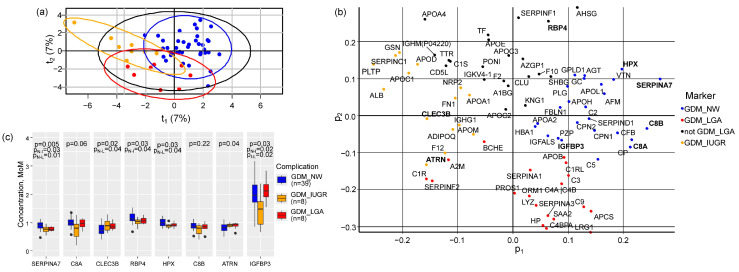
Multivariate analysis of first-trimester serum protein profiles in GDM subgroups. (**a**) PLS-DA score plot showing separation of patients with GDM + NW (blue), GDM + IUGR (orange), and GDM + LGA (red). Black circle is area of all samples location in score plot according standard deivation value across all samples. (**b**) Loading plot of proteins contributing to group discrimination. Proteins with variable importance in projection (VIP) > 1.5 are labeled in bold. (**c**) Comparative levels of selected protein markers across the three GDM subgroups. Box plots display median, interquartile ranges and outliers (dots). *p*-values indicate overall group differences (Kruskal–Wallis test). Pairwise comparisons were performed using Dunn’s test: p_N-I_ (GDM + NW vs. GDM + IUGR), p_I-L_ (GDM + IUGR vs. GDM + LGA), p_N-L_ (GDM + NW vs. GDM + LGA).

Partial least squares discriminant analysis (PLS-DA) was first used to compare three groups: GDM + NW (*n* = 39), GDM + IUGR (*n* = 8), and GDM + LGA (*n* = 8). The model explained 14% of the variation in serum protein levels (R^2^X = 0.14) and 52% of the differences between groups (R^2^Y = 0.52) (Figure 2a). Eight proteins emerged as key discriminators (variable importance in projection (VIP) > 1.5): Thyroxine-binding globulin (*SERPINA7*), complement components C8 alpha and beta chains (*C8A*, *C8B*), Tetranectin (*CLEC3B*), *RBP4*, Hemopexin (*HPX*), Attractin (*ATRN*), and *IGFBP3*. Each subgroup showed a distinct protein signature (Figure 2b). GDM + NW was characterized by elevated levels of *HPX*, *SERPINA7*, *C8B*, *C8A*, and *IGFBP3*. The GDM + IUGR group stood out with higher levels of *CLEC3B* and *ATRN*, while GDM + LGA was marked by lower *RBP4* levels. Pairwise comparisons confirmed these patterns. *SERPINA7* levels were significantly higher in GDM + NW than in either GDM + IUGR (*p* = 0.03) or GDM + LGA (*p* = 0.01). *CLEC3B* was lower in GDM + NW than in GDM + LGA. Both *RBP4* and *HPX* were higher in GDM + NW compared to GDM + IUGR (*p* = 0.04 for both) (Figure 2c).

**Figure 3:**
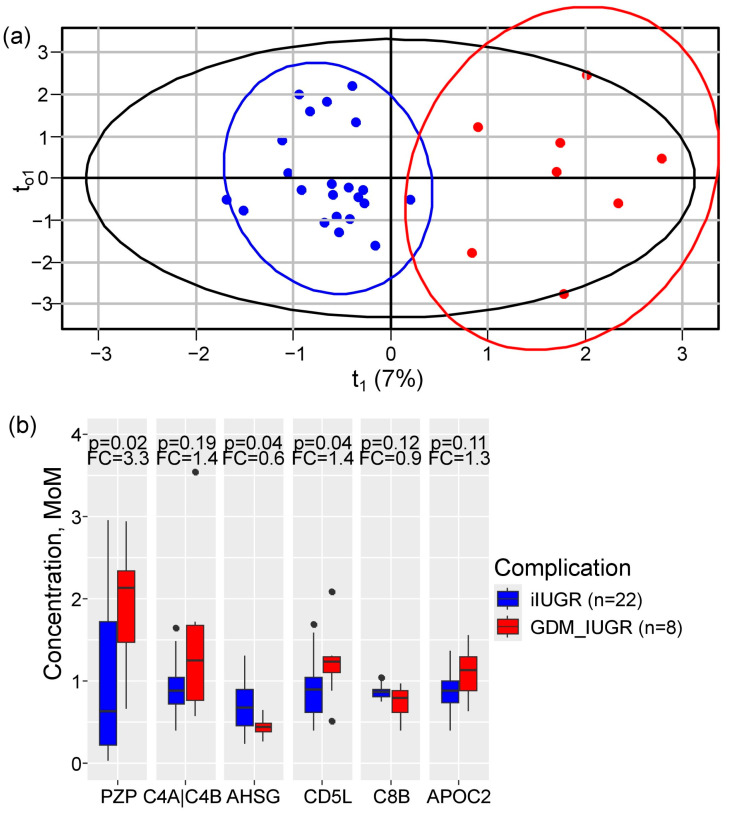
OPLS-DA distinguishes isolated IUGR from GDM + IUGR based on first-trimester serum protein profiles. (**a**) OPLS score plot showing separation of patients with isolated IUGR (blue) and GDM + IUGR (red). Black circle is area of all samples location in score plot according standard deivation value across all samples. (**b**) Comparative levels of key discriminative proteins between the two IUGR subgroups. Box plots display median, interquartile ranges and outliers (dots). *p*-values were calculated using the Mann–Whitney U test. Fold change (FC) represents the ratio of the median protein level in GDM + IUGR relative to isolated IUGR.

To determine whether isolated IUGR (*n* = 22) and IUGR complicated by GDM (*n* = 8) (GDM + IUGR) involve distinct molecular pathways—and consequently different first-trimester serum protein profiles—orthogonal partial least squares (OPLS) discriminant analysis was performed. The analysis revealed a clear separation between the two groups, explaining 19% of the variation in protein levels (R^2^X = 0.19) and 83% of the differences between them (R^2^Y = 0.83) (Figure 3a). Six proteins drove this separation (VIP > 1.5): *PZP*, *C4A|C4B*, *AHSG*, CD5 antigen-like (*CD5L*), *C8B*, and Apolipoprotein C-II (*APOC2*). Notably, the GDM + IUGR group exhibited marked differences compared to isolated IUGR: *PZP* increased 3.3-fold (*p* = 0.02) and *CD5L* increased 1.4-fold (*p* = 0.04), while *AHSG* showed a dramatic 94% reduction (*p* = 0.04) (Figure 3b).

**Figure 4:**
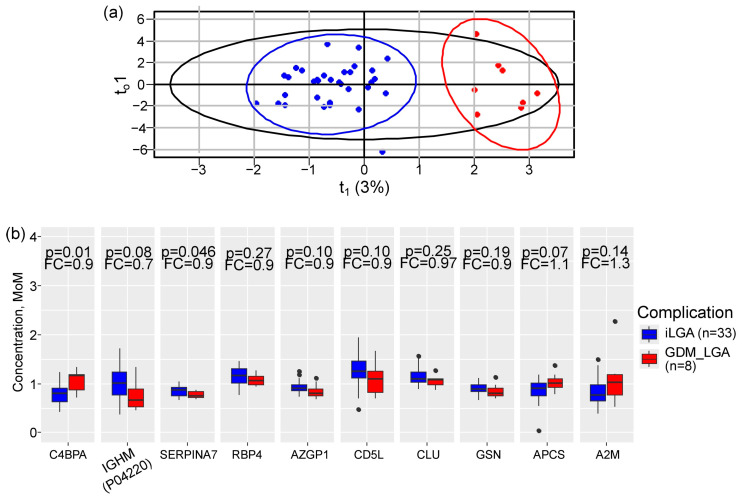
OPLS-DA distinguishes isolated LGA from GDM + LGA based on first-trimester serum protein profiles. (**a**) OPLS score plot showing separation of patients with isolated LGA (blue) and GDM + LGA (red). Black circle is area of all samples location in score plot according standard deivation value across all samples. (**b**) Comparative levels of key discriminative proteins between the two LGA subgroups. Box plots display median, interquartile ranges and outliers (dots). *p*-values were calculated using the Mann–Whitney U test. Fold change (FC) represents the ratio of the median protein level in GDM + LGA relative to isolated LGA.

Isolated LGA (*n* = 33) and GDM + LGA (*n* = 8) were then compared using OPLS analysis. The model explained 10% of protein variation (R^2^X = 0.10) and 81% of between-group differences (R^2^Y = 0.81) (Figure 4a). Eleven proteins contributed to this discrimination (VIP > 1.5), including C4b-binding protein alpha chain (*C4BPA*), Ig mu heavy chain disease protein and Ig mu chain C (*IGHM* [P04220]), *SERPINA7*, *RBP4*, Zinc-alpha-2-glycoprotein (*AZGP1*), *CD5L*, Clusterin (*CLU*), Gelsolin (*GSN*), Amyloid P component (*APCS*), and Alpha-2-macroglobulin (*A2M*). In GDM + LGA compared to isolated LGA, *C4BPA* increased 1.5-fold (*p* = 0.01), while *SERPINA7* showed a modest but significant decrease (10% reduction, *p* = 0.046) (Figure 4b).

**Figure 5:**
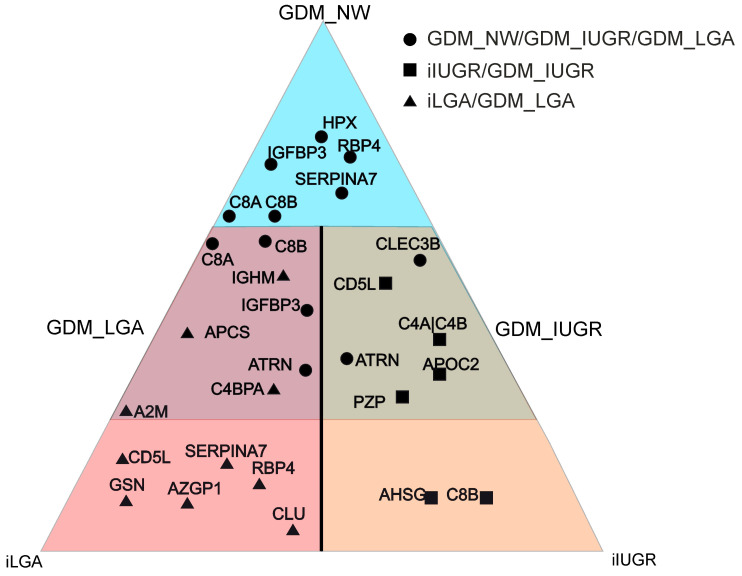
Protein markers with differential expression across subgroup comparisons. Only markers with increasing levels in a particular group are presented. Proteins shown include both those specific to individual comparisons and those emerging as discriminative markers in multiple multivariate models, demonstrating both unique and overlapping molecular signatures across different pregnancy complication phenotypes.

While most discriminative proteins were unique to specific comparisons, several appeared in multiple contexts (Figure 5). *C8B* consistently showed lower levels in GDM + IUGR, whether compared to isolated GDM, GDM + LGA, or isolated IUGR. *CD5L* played opposing roles: lower in isolated IUGR than in GDM + IUGR, but higher in isolated LGA than in GDM + LGA. Both *RBP4* and *SERPINA7* followed similar patterns—elevated in isolated GDM compared to either GDM + IUGR or GDM + LGA and also elevated in isolated LGA compared to GDM + LGA. These overlapping protein signatures, detectable as early as the first trimester, suggest shared and distinct pathological pathways underlying these pregnancy complications.

### 2.4. Pathway Enrichment Analysis of Discriminative Protein Markers

**Figure 6:**
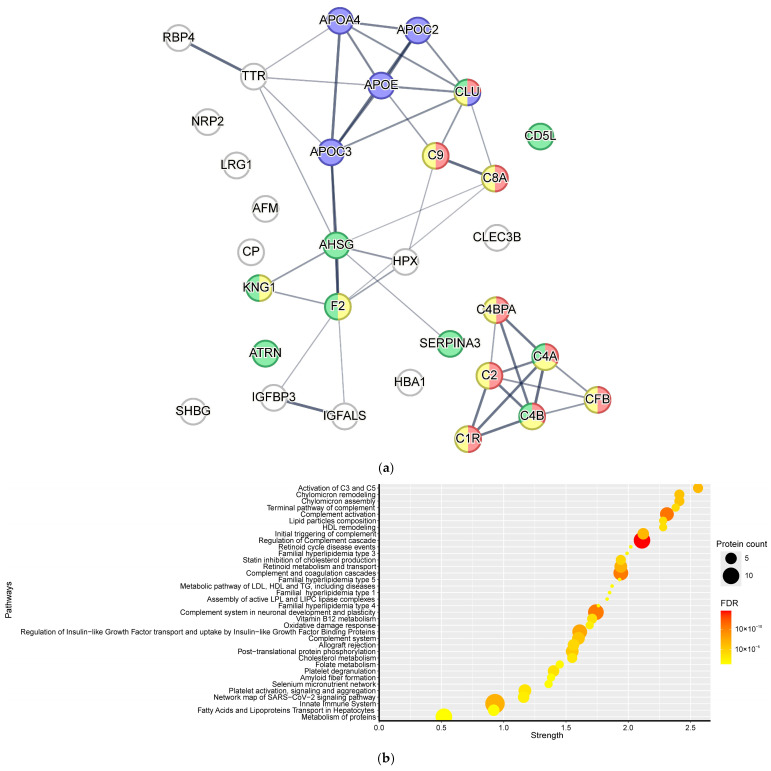
Protein–protein interaction network and pathway enrichment analysis. (**a**) Interaction network of proteins with significantly altered levels (*p* < 0.05) across the four clinical groups. Nodes are color-coded by functional annotation: red—complement activation, purple —reverse cholesterol transport, yellow—humoral immune response, green—inflammatory response, non color – wasn’t included in the largerscale biological process, which was statistical significant enriched by proteins markers. Edge thickness represents the confidence score of protein–protein interactions. (**b**) Significantly enriched pathways among the differentially expressed proteins (*p* < 0.05), based on Reactome and WikiPathways databases.

The 31 proteins that varied across the four clinical groups participate in a wide range of biological processes and metabolic pathways (Figure 6a, Table S9). When mapped to the Reactome and WikiPathways databases, these proteins enriched 35 distinct pathways. Among them, 12 pathways were related to energy metabolism—primarily cholesterol and triglyceride transport and synthesis—while 8 pathways were associated with the complement system (Figure 6b, Table S9).

Among the proteins discriminating isolated GDM, GDM + IUGR, and GDM + LGA, complement components *C8A* and *C8B* showed significant enrichment in the Terminal pathway of complement (Reactome) and complement activation (WikiPathways) (Table S10, Figure S3a).

For the proteins that separated isolated IUGR from GDM + IUGR, five of the six enriched pathways centered on complement metabolism. These included regulation of the complement cascade (driven by *C8B*, *C4A*|*C4B*), Activation of *C3* and *C5*, and initial triggering of complement (both driven by *C4A*|*C4B*) from the Reactome database. From WikiPathways, complement activation and complement system in neuronal development and plasticity were also enriched (Table S11, Figure S3b).

### 2.5. First-Trimester Predictive Models for LGA and IUGR Using Clinical and Proteomic Data

To evaluate the prognostic potential of first-trimester markers, predictive models for LGA and IUGR were developed using either clinical parameters or proteomic data. Several classification algorithms were tested, including OPLS-DA, support vector machines (SVM) with linear, polynomial, radial, and sigmoid kernels, random forest, and XGBoost. For each outcome, the optimal algorithm was selected based on the highest sum of sensitivity and specificity from 10-fold cross-validation, with Shapley values used to identify the most influential markers.

**Figure 7:**
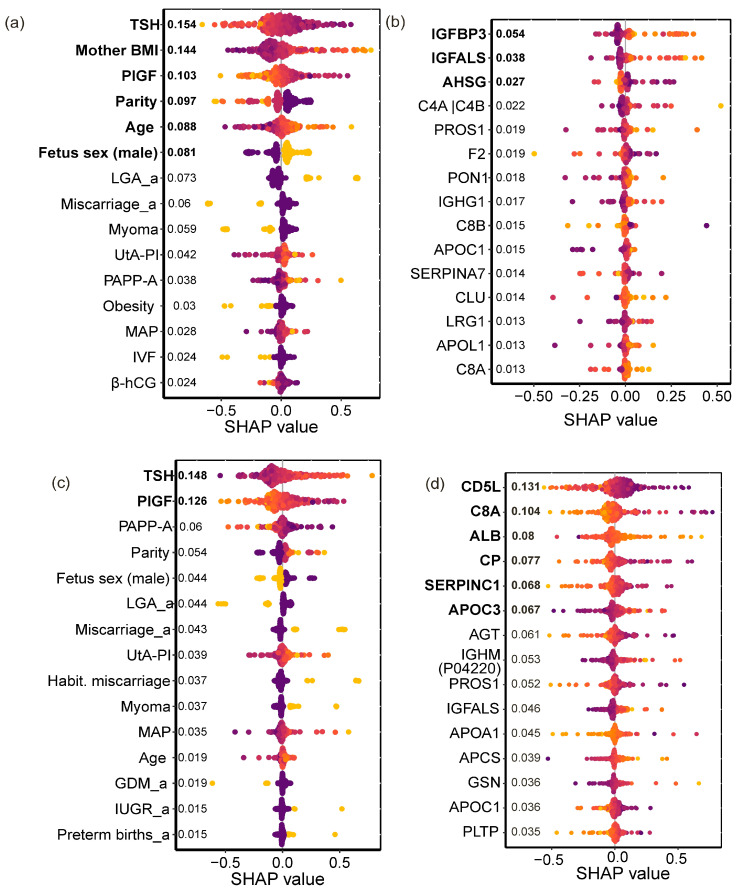
Key clinical and proteomic markers for discrimination between LGA and IUGR. Shapley values showing the 15 most influential features in the optimal models. Features are ranked by importance, with bold labels indicating markers that met the predefined threshold (Shapley value ≥ half of the maximum). Color indicates feature value: yellow—highest, purple—lowest. (**a**) Clinical markers for LGA discrimination. (**b**) Proteomic markers for LGA discrimination. (**c**) Clinical markers for IUGR discrimination. (**d**) Proteomic markers for IUGR discrimination. Clinical parameters measured during first-trimester screening included: TSH, PlGF, PAPP-A, mean arterial pressure (MAP), β-hCG, and uterine artery pulsatility index (UtA-PI). Obstetric history variables (denoted by “_a”) included: previous LGA infant (LGA_a), miscarriages (miscarriage_a), GDM (GDM_a), IUGR (IUGR_a), and preterm birth (preterm birth_a).

Using clinical parameters, an SVM model with a polynomial kernel (degree = 3.7, γ = 4.6 × 10^−5^, coef_0_ = 3.3 × 10^2^) identified women who later delivered LGA infants with 73% sensitivity and 78% specificity (Table 1). The key clinical markers for LGA prognosis were higher maternal BMI and age, lower TSH and PlGF serum levels, lower parity, and male fetal sex (Figure 7a). These findings align with earlier observations from this study: BMI was significantly higher in iLGA compared to physiological pregnancies and iIUGR, while TSH was significantly lower in GDM + LGA compared to GDM with normal fetal weight and GDM + IUGR.

Using first-trimester serum proteomic data, an SVM model with a radial kernel (γ = 1.9 × 10^−3^) predicted LGA with even higher performance: 88% sensitivity and 72% specificity. Increasing levels of *IGFBP3* and *IGFALS*, together with decreasing *AHSG*, emerged as the key proteomic markers for LGA prognosis (Figure 7b). Consistent with this, earlier analyses identified elevated *IGFBP3* and *IGFALS* as markers of GDM + LGA, while higher *AHSG* characterized isolated IUGR.

For IUGR prognosis, an SVM model using clinical parameters with a polynomial kernel (degree = 3.5, γ = 1.7 × 10^−6^, coef_0_ = 1.0 × 10^2^) achieved 77% sensitivity and 81% specificity. Higher TSH and lower PlGF levels were identified as the key clinical markers for predicting IUGR development (Figure 7c). These results corroborate earlier findings: TSH was significantly elevated in isolated IUGR compared to controls and iLGA, and in GDM + IUGR compared to GDM with normal fetal weight and GDM + LGA (*p* < 0.001 for all). Additionally, PlGF was significantly lower in iIUGR than in physiological pregnancy (*p* < 0.001), GDM (*p* = 0.001), and iLGA (*p* = 0.03).

Using proteomic data, SVM models with a linear and polynomial kernel predicted IUGR with 37% sensitivity and 84% specificity—comparable to the clinical model but with more limited sensitivity. Increasing levels of serum albumin (*ALB*) and apolipoprotein C-III (*APOC3*), together with decreasing levels of *CD5L, C8A*, ceruloplasmin (*CP*), and antithrombin-III (*SERPINC1*), were selected as proteomic markers for IUGR prognosis (Figure 7d). These findings are consistent with earlier observations: decreased *CD5L* characterized isolated IUGR compared to GDM + IUGR, while increased *C8A* was a marker of GDM + NW.

## 3. Discussion

This study presents a comprehensive analysis of first-trimester clinical parameters and serum proteomic profiles in pregnant women with different fetal growth abnormalities. Our findings demonstrate that isolated forms of IUGR and LGA, as well as their combination with GDM, represent not merely clinical variations but pathophysiologically distinct conditions. These can be differentiated as early as the first trimester using a combination of standard biochemical markers, clinical data, and in-depth quantitative serum proteomic analysis.

Analysis of obstetric history confirmed two distinct trajectories leading to growth pathology. In the isolated IUGR group, the high frequency of previous miscarriages and prior IUGR points to the role of chronic maternal factors, likely related to endothelial dysfunction and thrombophilia—a finding consistent with the theory of impaired deep trophoblast invasion [9,13]. In contrast, in the macrosomia groups (both isolated and GDM-associated), the key clinical predictors were maternal BMI and a history of macrosomia, highlighting the contribution of constitutional characteristics and/or undiagnosed metabolic disturbances [10].

Our data confirm the critical role of first-trimester placental factors. The significant reduction in PAPP-A and PlGF, accompanied by elevated TSH in women who subsequently developed IUGR (both isolated and GDM-associated), provides compelling evidence for early placental insufficiency [11,14]. PAPP-A and PlGF, both produced by the syncytiotrophoblast, are established markers of placental function [13]. Their reduction in the first trimester is known to predict IUGR and preeclampsia, reflecting impaired trophoblast invasion and subsequent placental insufficiency. Elevated TSH, even within the normal range, may also indicate early placentation disorders and an increased risk of IUGR [14].

The GDM + IUGR subgroup emerged as a particularly severe phenotype, characterized by the most extreme growth restriction and earliest delivery. This was underpinned by a marked reduction in first-trimester PlGF, suggesting a profound and early placental insult. This biochemical profile likely reflects a deleterious synergy of pathological processes: an underlying early placental insufficiency is further aggravated by the metabolic disturbances of GDM. In this context, GDM appears not merely as a comorbid condition but as an active exacerbator of placental dysfunction. The resulting environment is one of fetal hypoxia and undernutrition, a paradox given the maternal hyperglycemia, indicating a failure of nutrient and oxygen transfer despite increased substrate availability. This pathophysiological cascade is corroborated by the hemodynamic findings. Both iIUGR and GDM + IUGR groups exhibited the highest umbilical artery pulsatility indices and the lowest cerebroplacental ratios, classic signs of elevated placental vascular resistance and fetal circulatory centralization—the brain-sparing effect—in response to chronic hypoxia [15,16]. However, the severity was amplified in the GDM + IUGR group, aligning perfectly with the “double hit” concept. Here, the pre-existing placental insufficiency (first hit) [17] creates a vulnerability that is then amplified by the metabolic and inflammatory stress of GDM (second hit) [18]. This synergistic interaction accelerates placental decompensation, resulting in the most severe growth restriction and necessitating the earliest deliveries observed in our cohort [17,19]. This finding underscores that GDM with IUGR is not a simple combination of two common conditions but a unique, high-risk entity with a distinct pathophysiology.

A distinctly different hemodynamic and endocrine profile emerged in pregnancies resulting in fetal macrosomia. In the isolated LGA (iLGA) group, low first-trimester TSH levels were observed, likely reflecting the high metabolic state associated with excessive fetal growth [12]. This was accompanied by low umbilical artery resistance, indicative of robust, even excessive, placental perfusion. This hemodynamic pattern correlates with the increased placental mass and surface area characteristic of macrosomia, facilitating enhanced nutrient delivery to the growing fetus—a profile sometimes described as a “lazy” flow pattern due to the low resistance in a large, well-vascularized placental bed [20,21].

However, a critical divergence was observed when LGA occurred in the context of GDM. Compared to the iLGA group, the GDM + LGA cohort demonstrated a significant increase in umbilical artery resistance. Such hemodynamic alteration may represent an early sign of developing endothelial dysfunction within the fetoplacental unit. We hypothesize that chronic hyperglycemia superimposes microangiopathic changes on the placental vessels, similar to the vascular damage seen in other diabetic complications [22,23]. The resulting picture is a paradoxical state: although the fetus is born large—a result of early metabolic stimulation—its growth trajectory is accompanied by emerging maternal endothelial dysfunction [24] and a subclinical, yet elevated, risk of placental decompensation. These observations carry clinical importance, as they may explain the increased risk of adverse outcomes, including antenatal fetal demise, even in pregnancies that appear to be uncomplicated based on birth weight alone [25].

The proteomic analysis not only corroborated our clinical observations but also revealed specific molecular pathways involved in the pathogenesis of each phenotypic group. Complement proteins (*C4A|C4B*, *C8A*, *C8B*, *C4BPA*, *CD5L*) emerged as key discriminators in nearly all comparisons. The marked reduction in *C4A|C4B* level in isolated IUGR, with further alterations in GDM + IUGR, may reflect either complement consumption in the setting of chronic inflammation and endothelial damage, or genetically determined variations [26]. Conversely, elevated *C4BPA* levels in GDM + LGA may indicate activation of the lectin pathway of complement in response to metabolic stress and lipotoxicity [27]. These findings underscore the role of innate immunity and low-grade inflammation as a common denominator in the pathogenesis of pregnancy complications, manifesting with distinct directionality and magnitude depending on the specific phenotype.

The transport proteins *RBP4* and *SERPINA7* (thyroxine-binding globulin) emerged as markers of “metabolic well-being” in GDM with normal fetal weight, but their levels decreased in combined phenotypes. Reduced *RBP4*, a retinol transporter, may be related to increased tissue uptake or impaired liver/adipose tissue function in the context of decompensated diabetes [28]. The decrease in *SERPINA7* likely reflects alterations in thyroid status, which are known to be associated with placental dysfunction and impaired fetal growth—consistent with our first-trimester TSH findings [29].

Proteins of the IGF system (*IGFBP3, IGFALS*) proved to be robust markers of macrosomia. Their levels were significantly elevated in both isolated LGA and GDM + LGA groups, and they ranked among the top predictors for large-for-gestational-age neonates (Figure 7c). I*GFBP3*, the primary carrier of IGF-1 in circulation, prolongs its half-life and modulates its receptor availability [30,31]. Elevated *IGFBP3* and *IGFALS* in the first trimester likely reflect a highly anabolic metabolic state and placental preparation for enhanced nutrient transport, ultimately leading to excessive fetal growth [32].

From a clinical perspective, the most valuable models were those differentiating isolated from combined forms. Increased *PZP* and *CD5L* in GDM + IUGR compared to isolated IUGR may reflect enhanced immunomodulation and inflammatory response characteristic of diabetes [33,34]. Reduced *AHSG* in the same group is likely related to its role in inhibiting the insulin receptor and regulating calcification [35]; its deficiency may exacerbate vascular dysfunction [36].

Elevated *C4BPA* coupled with reduced *SERPINA7* and *RBP4* emerged as the key features distinguishing GDM + LGA from isolated LGA. This distinct molecular signature corroborates our hypothesis that macrosomia superimposed on GDM is characterized by subclinical inflammation and endothelial dysfunction, aligning this condition with more adverse pregnancy phenotypes.

Our data revealed that complement proteins (*C4A|C4B*, *C8A*, *C8B*, *C4BPA*, *CD5L*, *C1R*, *C2*) are key discriminators in IUGR. Emerging research has uncovered their critical role extending far beyond immune defense [37,38]. The complement system appears to be a major “architect” of the developing brain, performing three essential functions: progenitor cell proliferation, neuronal migration, and synaptic pruning. Complement components stimulate the proliferation of cells that will eventually form neurons. They guide the migration of young neurons to their final positions in the cerebral cortex. Complement proteins also “tag” weak, unnecessary synapses for removal by microglial cells—a critical process of neural network refinement during development [39]. Reduced levels of *C4A|C4B, C2, C1R* and *C8A* in maternal blood during IUGR may reflect their dysregulated expression or consumption within the fetal central nervous system. If synaptic pruning proceeds abnormally—either excessively or insufficiently—this can lead to the formation of incorrect neural connections, potentially manifesting later as autism spectrum disorders, schizophrenia, or epilepsy [40,41].

Alterations in the IGF system and oxidative stress markers are also directly relevant to brain development. Insulin-like growth factor 1 (IGF-1), carried by *IGFBP3* and *IGFALS*, is a potent neurotrophic factor. Studies in cell cultures and knockout mice have demonstrated that IGF-1 is essential for neuronal proliferation, maturation, differentiation, and protection from apoptosis [42,43]. A recent study in Nature (2025) using human embryos and organoids provided direct evidence that microglia stimulate proliferation of GABAergic neuron progenitors specifically through IGF-1 [44]. This discovery establishes a direct link between the IGF system and human brain development. Disruption of the IGF system, as observed in fetal growth disorders, may directly impact the formation of inhibitory neural circuits, whose deficiency underlies many neuropsychiatric conditions including anxiety and schizophrenia.

The application of machine learning methods (in particular, SVM) enabled the development of effective predictive models. Notably, clinical data demonstrated the highest sensitivity for predicting IUGR (77%), while proteomic data excelled in predicting LGA (88%). This may reflect the fact that molecular mechanisms underlying systemic metabolic changes (IGF and complement systems) have an early onset and can be detected at first-trimester screening. In contrast, the cascade of molecular alterations in the systemic circulation resulting from placental dysfunction and IUGR development may manifest later, after compensatory mechanisms of the placental unit become exhausted [45].

The key proteins identified in the optimal models by SHAP analysis align perfectly with our discriminative analyses. For LGA prediction, elevated *IGFBP3* and *IGFALS* with reduced *AHSG* emerged as key features. For IUGR prediction, increased *ALB* and *APOC3*, together with decreased *CD5L, C8A, CP*, and *SERPINC1*, were identified. Reduced *CP* and *SERPINC1* may indicate depletion of antioxidant and anticoagulant systems in the setting of placental insufficiency, further exacerbating endothelial damage.

This study has several notable strengths. First, its prospective design with first-trimester sample collection allowed for the investigation of early predictive markers before the clinical manifestation of pregnancy complications. Second, the inclusion of four distinct clinical groups—isolated IUGR, isolated LGA, and their combinations with GDM—enabled direct comparison of isolated versus combined phenotypes, revealing that these represent pathophysiologically distinct entities rather than simple clinical variations. Third, the integration of multiple data modalities—clinical parameters, routine biochemical markers, Doppler ultrasound, and in-depth quantitative proteomic analysis—provided a comprehensive view of the molecular and hemodynamic changes associated with each phenotype. Fourth, the application of advanced machine learning approaches (SVM with optimized hyperparameters) and SHAP value analysis allowed for robust model development and identification of the most influential predictors. Fifth, the use of first-trimester routine screening samples enhances the translational potential of our findings, as these samples are already collected in standard clinical practice.

Several limitations should be acknowledged. The relatively modest sample size, particularly within the GDM + IUGR and GDM + LGA subgroups, may limit the statistical power for detecting smaller differences and the generalizability of our predictive models. Although internal validation was performed using cross-validation, external validation in independent cohorts is necessary to confirm the robustness and reproducibility of our findings. The proteomic analysis followed ICH guidelines for Bioanalytical Method Validation, while comprehensive, was restricted to 85 serum proteins; a broader proteomic coverage might reveal additional discriminatory markers. Additionally, the observational nature of the study precludes establishing causal relationships between the identified protein alterations and the development of pregnancy complications. Finally, the clinical utility of the predictive models requires further evaluation in prospective implementation studies to assess their impact on clinical decision-making and pregnancy outcomes.

Despite these limitations, our findings provide a strong foundation for understanding the early molecular signatures of fetal growth abnormalities and highlight the potential for first-trimester risk stratification using routine clinical data and targeted serum proteomic analysis.

## 4. Materials and Methods

### 4.1. Study Design

This prospective cohort study was carried out at the V. I. Kulakov National Medical Research Center for Obstetrics, Gynecology and Perinatology between January and December 2022. The primary aim was to evaluate the relationship between first-trimester clinical and anamnestic data, routine biochemical markers, and the serum proteomic profile with the subsequent development of fetal growth disorders, specifically macrosomia and IUGR. A total of 1869 women aged 18 to 45 years were initially recruited during their routine first-trimester screening, performed in accordance with Fetal Medicine Foundation (FMF) guidelines between 11^+1^ and 14^+1^ weeks of gestation. Exclusion criteria included pre-existing diabetes mellitus, autoimmune disorders, a history of organ transplantation, malignancies, or fetal chromosomal abnormalities. After applying these criteria, a final cohort of 1720 women with singleton pregnancies was established.

To enhance analytical accuracy, a nested case–control design was implemented within this cohort. Case groups were formed based on the development of specific pregnancy complications: GDM (*n* = 55), iLGA, also referred to as isolated macrosomia (*n* = 33), and iIUGR (*n* = 22). Within the GDM group, 8 cases were also complicated by macrosomia and 8 by IUGR.

GDM was diagnosed using an oral glucose tolerance test (OGTT) conducted after an 8–14 h fast. Fasting plasma glucose was measured first; a diagnosis of GDM was established if the fasting level exceeded 5.1 mmol/L. In cases where fasting glucose was within the normal range, participants ingested 75 g of glucose dissolved in 200–300 mL of water. Plasma glucose levels were then reassessed at 1 and 2 h post-load. GDM was diagnosed if the 1 h glucose concentration surpassed 10.0 mmol/L or the 2 h level exceeded 8.5 mmol/L. Macrosomia was defined as a neonatal birth weight above the 90th percentile, while IUGR was defined as a birth weight below the 10th percentile for gestational age at delivery [45,46].

A control group of 83 women was selected from those with uncomplicated singleton pregnancies. Strict exclusion criteria for controls included conception via assisted reproductive technologies, existence/development of any hypertensive disorder, GDM, preterm birth before 37 weeks, macrosomia or IUGR (defined as an estimated fetal weight below the 10th percentile). This rigorously defined control group was recently utilized to establish pregnancy-specific reference intervals for 101 thoroughly validated serum proteins [47].

All participants provided written informed consent upon enrollment. A standardized first-trimester assessment was subsequently performed, including measurements of maternal weight, height, and blood pressure [48]. Ultrasound examination included transabdominal color Doppler assessment of the uterine artery pulsatility index (UtA-PI) [49]. Venous blood samples were collected into Serum Z/9 tubes (Monovette, Sarstedt, Germany) and processed within two hours of collection. After centrifugation at 300× *g* for 20 min at room temperature, the resulting serum supernatant was aliquoted into vials. Concentrations of placental growth factor (PlGF) and pregnancy-associated plasma protein-A (PAPP-A) were measured using the Delfia Xpress system (PerkinElmer, Shelton, CT, USA) strictly following the manufacturer’s protocol. Residual serum from these analyses was aliquoted into cryo-tubes and stored at −80 °C for subsequent targeted proteomic analysis of 139 first-trimester biomarkers [47].

The study protocol was reviewed and approved by the Institutional Review Board of the National Medical Research Center for Obstetrics, Gynecology and Perinatology (protocol No. 2, dated 9 March 2017). All procedures were conducted in full compliance with the ethical principles outlined in the Declaration of Helsinki and the standards of Good Clinical Practice.

### 4.2. Sample Preparation

A targeted proteomic assay employing 85 stable isotope-labeled (SIS) internal standards and their unlabeled (NAT) synthetic counterparts was used in this study. Peptide synthesis and characterization were performed at Skoltech following established protocols [50,51], with the assay adapted from the BAK-270 kit (MRM Proteomics Inc., Montreal, QC, Canada) [47,52].

Sample preparation was carried out according to standard procedures [47,50,53]. Briefly, 10 µL of serum was denatured in 9 M urea with 20 mM dithiothreitol (30 min, 37 °C), alkylated with 100 mM iodoacetamide (30 min, dark), and digested overnight with TPCK-trypsin (20:1 ratio, 18 h, 37 °C). Digestion was stopped with formic acid (1% final, pH ≤ 2), yielding ~1 mg/mL peptide concentration. Following solid-phase extraction (SPE), peptides were reconstituted in 34 µL of 0.1% FA for LC-MRM-MS analysis.

The SIS peptide mixture was spiked into all clinical samples (*n* = 193), calibration standards, and quality controls (QCs) at 100 fmol/µL. NAT peptides were added only to the seven-point BSA-based calibration curve (levels A–G), spanning the lower to upper limits of quantification. Quality control included BSA-based QCs at low, medium, and high concentrations (QCA–C). Process blanks were analyzed to assess potential background contamination. All calibration standards and QCs were processed identically to clinical samples.

### 4.3. LC-MRM-MS Analysis

LC-MRM-MS analysis was conducted across three randomized batches using an ExionLC UHPLC system (ThermoFisher Scientific, Waltham, MA, USA) coupled to a SCIEX QTRAP 6500+ mass spectrometer (SCIEX, Toronto, ON, Canada). All 193 clinical specimens were measured in duplicate, with random allocation across batches. Each 96-well plate contained a complete seven-point calibration curve (A-G), BSA-based QCA–C, and process blanks to ensure analytical integrity [47,51,52].

Chromatographic separation was achieved on a C18 column (2.1 × 150 mm, 1.7 µm) using a linear gradient of 2–45% acetonitrile in 0.1% formic acid at 0.4 mL/min. The mass spectrometer operated in positive electrospray ionization mode (4000 V source voltage, 450 °C). Data were acquired via multiple reaction monitoring; precursor/product ion transitions are listed in Table S12.

Quality assurance was embedded in the run design: calibration curves were analyzed at the start of each batch, while QC samples were positioned at the beginning, middle, and end to continuously monitor system performance and reproducibility.

### 4.4. Data Processing

Raw mass spectrometry data were processed and quantified using Skyline software (version 20.2.0.343) [54]. Peptide concentrations (fmol/µL plasma) were derived from seven-point calibration curves (A–H) using a 1/x^2^-weighted linear regression model. Quantification and quality assessment followed ICH guidelines for Bioanalytical Method Validation [55].

Assay performance was evaluated using calibration standards and QC samples. Acceptance required accuracy and precision within ±20% of theoretical values. A calibration curve was accepted if at least five of seven points met these criteria. For batch validity, ≥66% of QC samples and ≥90% of peptide calibration curves had to pass. Proteins with calibration curve R^2^ > 0.99 were considered “quantified.”

Concentrations below the lower limit of quantification (LLOQ, level A) were imputed with the LLOQ value; those above the upper limit (ULOQ, level H) were assigned the ULOQ value. Proteins with >50% of study samples outside the quantifiable range were excluded from further analysis.

### 4.5. Statistical Analysis

Clinical parameters were compared across the four main study groups (control, isolated GDM, isolated IUGR, and isolated LGA) as well as across the three GDM subgroups (isolated GDM, GDM + IUGR, and GDM + LGA). For continuous variables, the Kruskal–Wallis test was used, while categorical variables were analyzed using the chi-square test, with statistical significance defined as *p* < 0.05. When significant differences were detected, post hoc pairwise comparisons were performed with appropriate adjustment for multiple testing.

For comparisons between two specific subgroups—isolated IUGR versus GDM + IUGR, and isolated LGA versus GDM + LGA—the Mann–Whitney U test was applied for continuous variables and the chi-square test for categorical variables. Statistical power was calculated for these pairwise comparisons to assess the reliability of the findings.

To correct for batch effects and analytical variability, data normalization was performed using a combination of the ComBat and RobNorm methods [56]. For each protein, the mean CV was calculated across standard and quality control samples between batches; proteins with a CV exceeding 50% were excluded from further analysis. Protein expression values were then adjusted using MoM, derived from a previously established reference study [47], and subsequently corrected for maternal age, body mass index, parity, fetal sex, presence of uterine myomas, and gestational age at blood collection, based on models developed in the same prior work [47].

Initial comparisons were performed across four clinical groups: first group (control, *n* = 83), second group (GDM, *n* = 55), third group (iIUGR, *n* = 22), and fourth group (iLGA, *n* = 33). Group differences were assessed using the Kruskal–Wallis test with a significance threshold of *p* < 0.05. When significant, post hoc Dunn’s test was applied for pairwise comparisons.

Within the GDM cohort, patients were further stratified into three subgroups: isolated GDM, GDM with concomitant IUGR, and GDM with concomitant macrosomia. These subgroups were compared using orthogonal projections on PLS-DA [57], with protein markers selected based on a VIP score > 1.5. Levels of the identified potential markers were subsequently compared using the Kruskal–Wallis test.

To assess the impact of GDM on growth disorders, patients with IUGR (*n* = 30) and LGA (*n* = 41) were further stratified by the presence or absence of GDM: iIUGR (*n* = 22) versus IUGR with GDM (*n* = 8), and iLGA (*n* = 33) versus LGA with GDM (*n* = 8). Discrimination analyses were performed using OPLS-DA [58], with marker selection based on VIP > 1.5. Potential marker levels were compared between subgroups using the Mann–Whitney U test.

For each set of selected protein markers, protein–protein interaction networks and metabolic pathway enrichment analyses were conducted using the STRING database [59]. Pathways with a false discovery rate (FDR) below 0.05 were considered significantly enriched. An enrichment score was calculated as the weighted harmonic mean of the observed-to-expected protein ratio and −log(FDR).

Predictive models for LGA and IUGR were developed using either the proteomic dataset or clinical parameters alone. The following classification algorithms were employed: OPLS-DA [58], SVM with linear, polynomial, radial, and sigmoid kernels [60], Random forest [61], and XGBoost [62]. Hyperparameters for the non-linear SVM kernels and XGBoost were optimized using particle swarm optimization [63] with five repeats of 5-fold cross-validation. The optimal model was selected based on the highest sum of sensitivity and specificity, as determined by 10-fold cross-validation. For variables incorporated into each optimal model, Shapley values were computed [64]; features with Shapley values at least half of the maximum value were considered potential markers.

All statistical analyses were performed using R version 4.3.2 [65], the RStudio environment (version 2023.09.1) [66]. A comprehensive suite of R packages was employed to support both statistical modeling and data visualization. For modeling and machine learning tasks, the packages ropls 1.34.0 [67], effsize 0.8.1 [68], dunn.test 1.3.6 [69], jgsbook 1.0.7 [70], Desctools 0.99.60 [71], pwr 1.3-0 [72], xgboost 1.7.8.1 [62], e1071 1.7-16 [73], caret 7.0-1 [74], dplyr 1.1.4 [75] were utilized. Visualization of the results was accomplished using ggplot2 3.5.2 [76], reshape2 1.4.4 [77], forcats 1.0.0 [78], ggrepel 0.9.6 [79] and pROC 1.18.5 [80].

## 5. Conclusions

This prospective study demonstrates that isolated IUGR, isolated LGA, and their combinations with GDM represent pathophysiologically distinct entities with detectable first-trimester signatures. The GDM + IUGR phenotype emerges as the most severe “double hit” condition, characterized by the lowest PlGF levels, earliest delivery, and worst perinatal outcomes, while GDM + LGA, despite excessive fetal growth, shows evidence of underlying placental endothelial dysfunction. Proteomic profiling identifies complement proteins as central discriminators of fetal growth disorders and IGF system proteins as robust early markers of macrosomia. Machine learning models achieve 77% sensitivity for IUGR prediction using clinical parameters and 88% sensitivity for LGA prediction using proteomic data. Several limitations warrant consideration, including the modest sample sizes in combined phenotype subgroups which limit statistical power and generalizability, the targeted proteomic approach restricted to 80 serum proteins, and the observational design that precludes causal inferences. Despite these limitations, this study establishes that first-trimester routine screening samples can be leveraged for early risk stratification, laying the foundation for precision-based approaches to prenatal risk assessment.

## Figures and Tables

**Table 1:** Performance of predictive models for LGA and IUGR. For each outcome, multiple classification algorithms were tested using either clinical parameters or proteomic data. The optimal model for each outcome—selected based on the highest sum of sensitivity and specificity from 10-fold cross-validation—is indicated in bold.

Method	Pathology	Sensitivity	Specificity	Accuracy	Sensitivity + Specificity
**Clinical parameters**					
OPLS-DA	LGA	0.37	0.98	0.85	1.35
SVM, linear kernel		0.68	0.82	0.79	1.50
**SVM, polynomial kernel**		**0.73**	**0.78**	**0.77**	**1.51**
SVM, radial kernel		0.61	0.81	0.77	1.42
SVM, sigmoid kernel		0.66	0.67	0.67	1.33
Random Forest		0.17	0.97	0.80	1.14
Xgboost		0.49	0.90	0.81	1.39
OPLS-DA	IUGR	0.23	0.98	0.87	1.21
SVM, linear kernel		0.57	0.88	0.83	1.44
**SVM, polynomial kernel**		**0.77**	**0.81**	**0.80**	**1.58**
SVM, radial kernel		0.00	1.00	0.84	1.00
SVM, sigmoid kernel		0.83	0.59	0.63	1.42
Random Forest		0.13	0.99	0.85	1.12
Xgboost		0.60	0.75	0.73	1.35
**Proteomic data**					
OPLS-DA	LGA	0.39	0.90	0.79	1.29
SVM, linear kernel		0.34	0.80	0.70	1.14
SVM, polynomial kernel		0.83	0.75	0.77	1.58
**SVM, radial kernel**		**0.88**	**0.72**	**0.75**	**1.60**
SVM, sigmoid kernel		0.80	0.63	0.66	1.43
Random Forest		0.10	0.97	0.78	1.06
Xgboost		1.00	0.00	0.21	1.00
OPLS-DA	IUGR	0.17	0.96	0.83	1.12
**SVM, linear kernel**		**0.37**	**0.84**	**0.77**	**1.21**
**SVM, polynomial kernel**		**0.37**	**0.84**	**0.77**	**1.21**
SVM, radial kernel		0.00	1.00	0.84	1.00
SVM, sigmoid kernel		1.00	0.00	0.16	1.00
Random Forest		0.03	1.00	0.85	1.03
Xgboost		0.23	0.89	0.79	1.12

## Data Availability

The original contributions presented in this study are included in the article/Supplementary Materials. Further inquiries can be directed to the corresponding author.

## Supplementary Materials

The following supporting information can be downloaded at: https://www.mdpi.com/article/10.3390/ijms27104192/s1.

## Abbreviations

The following abbreviations are used in this manuscript:

BMI	Body mass index
CS	Cesarean section
CV	Coefficient of variation
GDM	Gestational Diabetes mellitus
FDR	False discovery rate
HLOQ	The highest limit of quantification
IUGR	Intrauterine growth restriction
LC-MS	Liquid chromatography-mass spectrometry
LGA	Large for Gestational Age
LLOQ	The lowest limit of quantification
MAP	Mean arterial pressure
MoM	Multiply of medians
MRM	Multiple reaction monitoring
MS	Mass spectrometry
NAT	Natural synthetic proteotypic peptides
(O)PLS-DA	(Orthogonal) projections on latent structure discriminant analysis
RF	Random Forest
SIS	Stable isotope-labeled standards
SVM	Support vector machine
TSH	Thyroid stimulating hormone
VIP	Variable importance in projection
QC	Quality control
XGBoost	Extreme Gradient boosting
