# Supplementary material for: Integrated Clinical and Molecular Profiling of Fetal Growth Disorders in the First Trimester
Source: Int J Mol Sci. 2026 May 8;27(10):4192. doi: 10.3390/ijms27104192 (PMC13206995; doi:10.3390/ijms27104192)
Supplement: Supplementary file 1 [file ijms-27-04192-s001.zip › Supplementary figure_040502026.pdf]

Figure S1. Heatmap of statistical significant alterated clinical parameters (red - increasing, blue - decreasing) (a) In case of GDM, isolated IUGR and isolated LGA comparing with control; (b) In case of IUGR with GDM and LGA with GDM comparing with normaweight GDM.

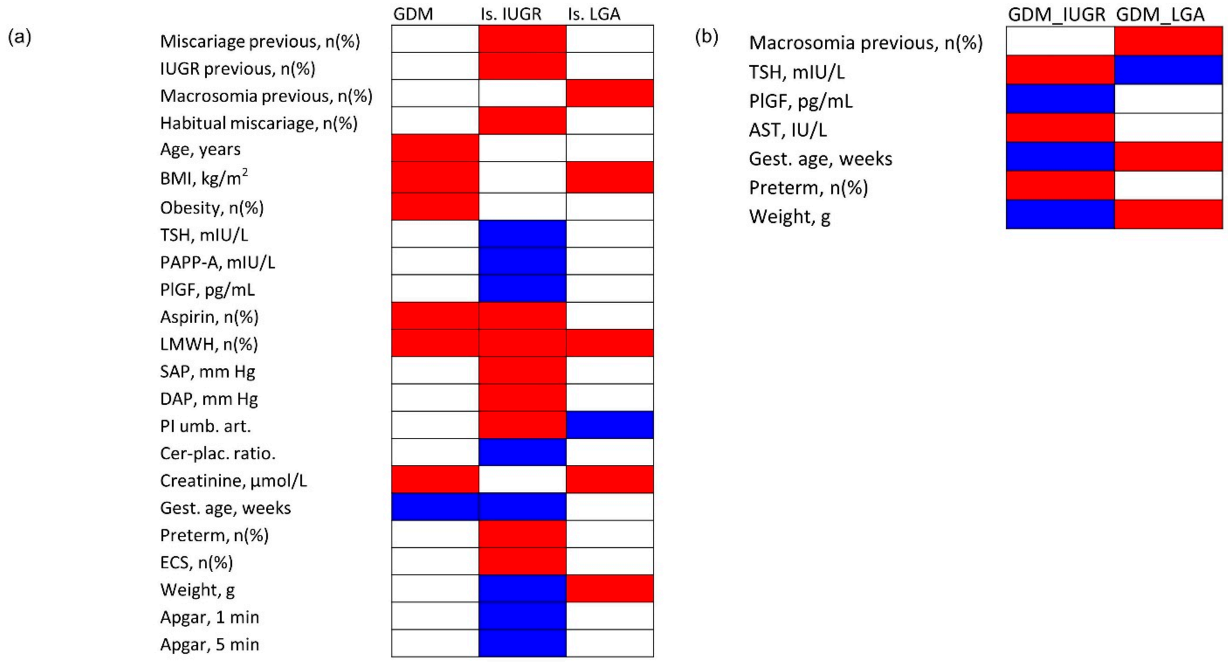

Figure S2. MRM-Proteome profile of serum samples after normalization and clinical parameters correction.

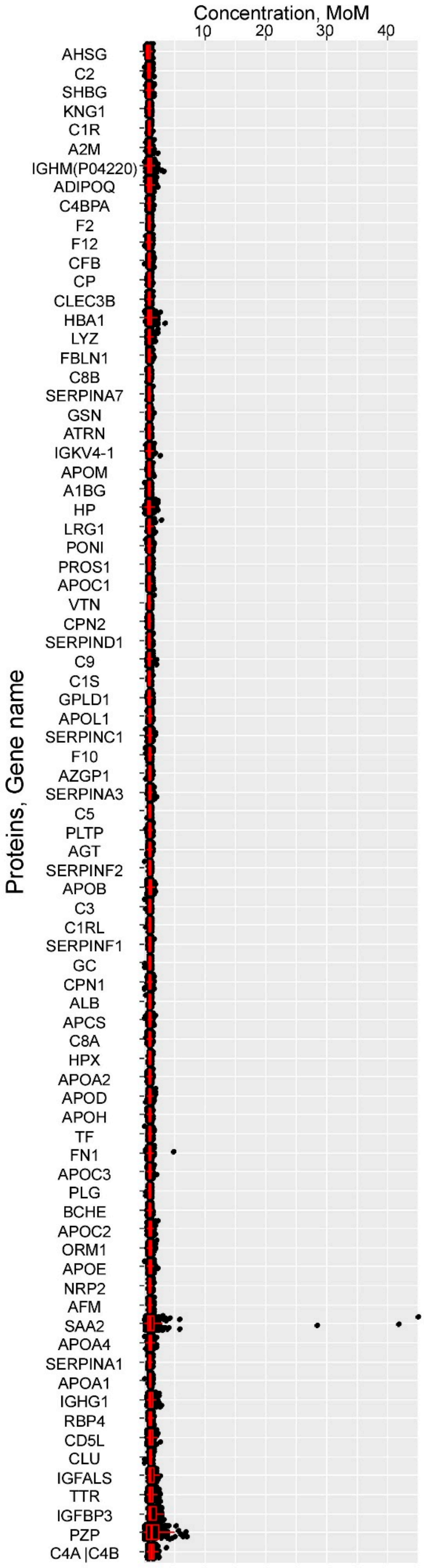

Figure S3. Statistical significant enriched pathways by markers of (a) isolated GDM/GDM coupled with IUGR/ GDM coupled with macrosomia discrimination; (b) isolated IUGR/ IUGR coupled with GDM discrimination.

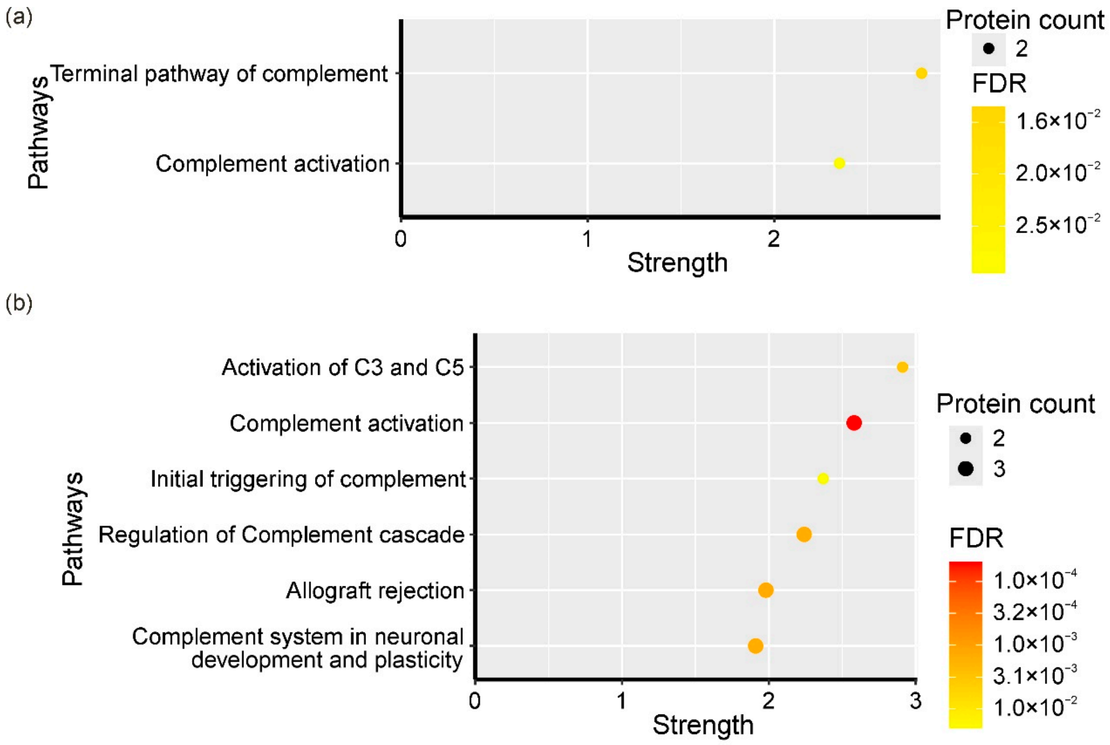

.
